# DAPK1 as an independent prognostic marker in liver cancer

**DOI:** 10.7717/peerj.3568

**Published:** 2017-07-19

**Authors:** Ling Li, Libin Guo, Qingshui Wang, Xiaolong Liu, Yongyi Zeng, Qing Wen, Shudong Zhang, Hang Fai Kwok, Yao Lin, Jingfeng Liu

**Affiliations:** 1 Hepatopancreatobiliary Surgery Department, The First Affiliated Hospital of Fujian Medical University, Fuzhou, Fujian Province, China; 2 United Innovation of Mengchao Hepatobiliary Technology Key Laboratory of Fujian Province, Mengchao Hepatobiliary Hospital of Fujian Medical University, Fuzhou, China; 3 Faculty of Health Sciences, University of Macau, Taipa, Macau; 4 College of Life Sciences, Fujian Normal University, Fuzhou, Fujian Province, China; 5 Centre for Cancer Research & Cell Biology, School of Medicine, Dentistry and Biomedical Sciences, Queen’s University Belfast, Belfast, United Kingdom; 6 Northern Ireland Centre for Stratified Medicine, Biomedical Sciences Research Institute, University of Ulster, Londonderry, United Kingdom

**Keywords:** Liver cancer, IHC, Survival, DAPK1, Prognosis

## Abstract

The death-associated protein kinase 1 (DAPK1) can act as an oncogene or a tumor suppressor gene depending on the cellular context as well as external stimuli. Our study aims to investigate the prognostic significance of DAPK1 in liver cancer in both mRNA and protein levels. The mRNA expression of DAPK1 was extracted from the Gene Expression Omnibus database in three independent liver cancer datasets while protein expression of DAPK1 was detected by immunohistochemistry in our Chinese liver cancer patient cohort. The associations between DAPK1 expression and clinical characteristics were tested. DAPK1 mRNA expression was down-regulated in liver cancer. Low levels of DAPK1 mRNA were associated with shorter survival in a liver cancer patient cohort (*n* = 115; *p* = 0.041), while negative staining of DAPK1 protein was significantly correlated with shorter time to progression (*p* = 0.002) and overall survival (*p* = 0.02). DAPK1 was an independent prognostic marker for both time to progression and overall survival by multivariate analysis. Liver cancer with the b-catenin mutation has a lower DAPK1 expression, suggesting that DAPK1 may be regulated under the b-catenin pathway. In addition, we also identified genes that are co-regulated with DAPK1. DAPK1 expression was positively correlated with IRF2, IL7R, PCOLCE and ZBTB16, and negatively correlated with SLC16A3 in both liver cancer datasets. Among these genes, PCOLCE and ZBTB16 were significantly down-regulated, while SLC16A3 was significantly upregulated in liver cancer. By using connectivity mapping of these co-regulated genes, we have identified amcinonide and sulpiride as potential small molecules that could potentially reverse DAPK1/PCOLCE/ZBTB16/SLC16A3 expression. Our study demonstrated for the first time that both DAPK1 mRNA and protein expression levels are important prognostic markers in liver cancer, and have identified genes that may contribute to DAPK1-mediated liver carcinogenesis.

## Introduction

The death-associated protein kinase 1 (DAPK1) is an important regulator for apoptosis, when dysregulated may cause cancer. Interestingly, DAPK1 can act as oncogene or tumor suppressor gene depending on the cellular context as well as external stimuli (Schneider-Stock, 2014; Steinmann et al., 2015). Down-regulation of DAPK1 expression through promoter methylation has been shown to be a prognostic factor in various types of cancer, including chronic lymphocytic leukemia (Raval et al., 2007), chronic myeloid leukemia (Qian et al., 2009), diffuse large B-cell lymphoma (Kristensen et al., 2014), cervical cancer (Feng et al., 2005), esophageal cancer (Brabender et al., 2009) and lung cancer (Feng et al., 2008). Mechanistically, DAPK1 has been shown to suppress tumors via multiple pathways such as inhibition of the oncogenic function of Pin1 (Lee et al., 2011). On the other hand, DAPK1 has also been shown to promote growth and is essential for survival for p53-mutated breast cancers (Zhao et al., 2015).

DAPK1 exerts its functions through various biological processes, including apoptosis (Deiss et al., 1995; Inbal et al., 1997), autophagy (Gade et al., 2012) and even metastasis (Inbal et al., 1997). The activity of DAPK1 has been shown to be regulated through Src kinase (Wang et al., 2007), the Ras/MEK/ERK pathway (Anjum et al., 2005; Chen et al., 2005) and the mTORC1 pathway (Lin et al., 2011). Although extensively researched, the role of DAPK1 and its prognostic significance in liver cancer is largely unknown.

DAPK1 promoter methylation was investigated previously by others in liver cancer. Promoter methylation was not detected in liver cancer specimens (Yu et al., 2002). On the other hand, DAPK1 has been shown to be a prognostic marker in a Japanese liver cancer patient cohort with a small sample size (Matsumoto et al., 2003). However, the mRNA expression levels of DAPK1 in liver cancer and the prognostic significance of protein and mRNA expression in Chinese liver cancer patients, to the best of our knowledge, have not been published. In this study, we aimed to use online available microarray data from the Gene Expression Omnibus (GEO) database and an in-house liver cancer patient cohort to study the prognostic significance of DAPK1 mRNA and protein expression, respectively, in liver cancer.

## Materials and Methods

### Extraction of clinical and microarray gene expression data from bladder cancer patient datasets

A search was performed using liver cancer/hepatocellular carcinoma in GEO database. Two datasets with more than 400 specimens in a single microarray platform, GSE25097 (Sung et al., 2012) and GSE36376 (Lim et al., 2013), were identified. In addition, a dataset, GSE76427 (not yet associated with a publication), with more than 100 hepatocellular carcinoma specimens as well as survival status was identified. Another dataset, GSE9843 (Villanueva et al., 2011), with gene signature subgrouping as well as gene mutation status was also identified. The data was extracted and analyzed.

### Patients and specimens

The study cohort was composed of liver specimens from 101 patients with hepatocellular carcinoma (87 men and 14 women, mean age: 50.7). The specimens were obtained from a curative resection scheduled between October 2002 and March 2011 at the Mengchao Hepatobiliary Hospital of Fujian Medical University. The Institution Review Board of the Mengchao Hepatobiliary Hospital of Fujian Medical University has approved the project (Ethical Application Ref: 2016_009_01). Prior to surgery, written consents were obtained from all participants. The diagnostic criteria of the American Association for the Study of Liver Diseases were used for clinical and pathological diagnosis. The clinical information for this China patient cohort is listed in Table 1.

**Table 1:** Patients’ clinical features. Patient clinical and pathologic features (*n* = 101).

	Number of cases	%	Median (range)
Age	101	100	51 (14–73) years
Tumor size	101	100	10 (2–24) cm
DAPK1 Staining			
Negative	44	43.6	
Positive	57	56.4	
Sex			
Female	14	13.9	
Male	87	86.1	
Cirrhosis			
Without	29	28.7	
With	72	71.3	
Differentiation			
Well	26	25.7	
Intermediate	58	57.4	
Poor	17	16.8	
Metastasis			
Without	26	25.7	
With	14	13.9	
Missing	61	60.4	

### Construction of tissue microarray

HCC specimens from patients (*n* = 101) were included in the tissue microarray constructed (Zhao et al., 2015) as previously described (Xu et al., 2014). Manual tissue arrayer was used to pick the tissue cores from selected areas. The tissue was then transferred to the corresponding location of the recipient block. Two areas from each specimen were selected.

### Immunohistochemistry and evaluation

Immunohistochemical staining of DAPK1 was performed using the two-step Envision plus staining technique. A 4 µm section was cut from the TMA block. The section was then de-paraffinised and rehydrated by xylene and graded ethanol, respectively. The specimens were then incubated in 0.01 mol/L sodium citrate buffer (pH 6.0) for 2 min and high-pressure antigen retrieval procedure was applied. Endogenous peroxidase activity was blocked by using 3% hydrogen peroxide. The specimens were incubated with rabbit anti-human DAPK1 (LS-C137867, 1:2500 dilution, LSBio, monoclonal) antibody in a humidified chamber at 4°C overnight. The specimens were washed with PBS, incubated with HRP-conjugated secondary antibody (ZSGB-BIO, Shanghai, China) for 30 min at room temperature, and washed again with PBS. The specimens were then subjected to diaminobenzidine colouration and were counterstained with 20% hematoxylin.

Two pathologists, who were blinded to the clinical parameters, reviewed and scored independently according to the intensity of staining as previously described (Sun et al., 2016). Re-evaluation for coming up with a consensus score was performed when there was discrepancy. The expression was considered as negative if the specimens had negative staining and was considered positive if the specimens were stained with light to strong brown color.

### Identification of DAPK1 co-expressing genes

The gene expression patterns of patients in DAPK1-low subgroup and those in the DAPK1-high subgroup were compared. Probesets that were differentially expressed between these two subgroups were identified by 2-sample Welch’s *T*-test using Java application MyStats. *P* values were used to rank for all the probesets available.

### Identification of potential therapeutic small molecule down-regulated DAPK1 and its co-regulated genes through connectivity mapping

Gene expression connectivity mapping was performed to identify candidate small molecule compounds that may reverse the reduced expression of DAPK1 and its associated gene expression signature using a statistically significant connections’ map (sscMap) as previously described (Lamb, 2007; Lamb et al., 2006; Zhang & Gant, 2009). The relevant probes were compared with the 6,000 gene expression profiles generated by treating cancer cells with over 1,000 small molecules (Zhang & Gant, 2009) with gene signature perturbation procedure applied as previously described (McArt & Zhang, 2011). All the small molecular compounds identified were sorted and ranked by their *p*-value, perturbation stability and standardized connection score.

### Statistical analysis

All statistical analyses were performed using SPSS19.0. Comparison of 2-by-2 categories was performed with the use of Fisher’s exact test. Time-to-progress/relapse or time-to-death comparisons were performed with the use of a log-rank test. Univariate Cox-regression and multivariate analysis was also performed for available clinico-pathological parameters. Multivariate Cox-regression analysis with forward stepwise selection and an entry limit of *p* < 0.05 was performed to identify independent predictors of survival in the patient cohort.

## Results

### DAPK1 mRNA expression is decreased in liver cancer

**Figure 1:**
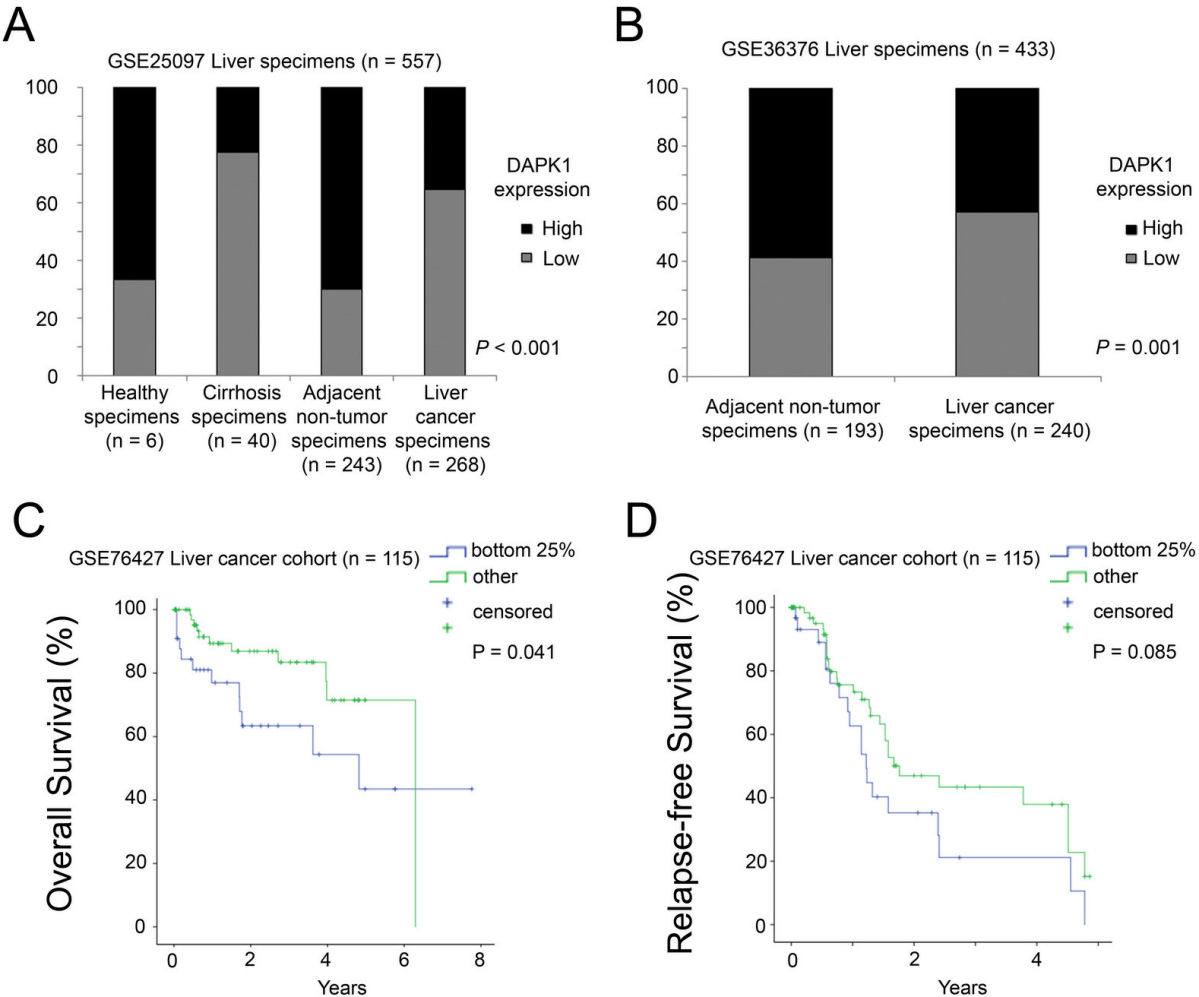
mRNA expression of DAPK1 in liver specimens. (A) A histogram showing the percentage of cases with different levels of DAPK1 mRNA expression in the GSE25097 dataset with four different types of liver specimens. (B) A histogram showing the percentage of cases with different levels of DAPK1 mRNA expression in the GSE36376 dataset with two different types of liver specimens. (C) Kaplan–Meier overall survival curves for patients with different levels of DAPK1 mRNA expression in liver cancer cohort GSE76427 (D) Kaplan–Meier relapse-free survival curves for patients with different levels of DAPK1 mRNA expression in liver cancer cohort GSE76427.

In the GSE25097 dataset, there were 557 specimens, of which six were from healthy donors, 40 from cirrhotic livers, 243 from adjacent non-tumor and 268 from liver cancer. We tested the DAPK1 expression in these specimens. Using the median expression as the cut-off point, we found that 67% of the healthy specimens and 70% of the adjacent non-tumor expressed DAPK1 at a high level, but only 23% and 35% of cirrhotic and tumor specimens, respectively, expressed DAPK1 at a high level. The difference was statistically significant (Chi-Square test, *p* < 0.001; Fig. 1A). The results suggest that DAPK1 is down-regulated in tumor/cirrhotic liver specimens compared to non-tumor and healthy liver specimens. To confirm this observation, we tested DAPK1 expression in another dataset, GSE36376, which consists of 193 adjacent non-tumor specimens and 240 liver cancer specimens. A high level DAPK1 expression was observed in 59% of non-tumor specimens but only 43% liver cancer specimens had similar level of DAPK1 expression (Fisher’s exact test, *p* = 0.001; Fig. 1B). The results suggest that DAPK1 expression was down-regulated in liver cancer.

### Decreased DAPK1 mRNA expression is associated with poor prognosis

Since survival data was not available in both the GSE25097 and GSE36376 datasets, another liver cancer dataset (GSE76427), with survival data available and a sample size of >100 patients was identified in the GEO database. As shown in Fig. 1C, using a lower quartile as the cut-off, patients with a relatively low level expression of DAPK1 had a mean survival time of only 4.5 years, which was significantly shorter compared to the mean survival time of 5.2 years in patients with a relatively high level expression of DAPK1 (log rank test, *p* = 0.041). Similar trend was observed for relapse-free survival. Patients with a relatively low level expression of DAPK1 had a mean relapse-free survival of 1.9 years, while those with a relatively high level expression of DAPK1 had a mean relapse-free survival of 2.6 years (log-rank test, *p* = 0.085; Fig. 1D). The results suggest that DAPK1 mRNA expression is a favorable prognostic marker for liver cancer patients.

### Decreased DAPK1 protein expression is associated with poor prognosis

**Figure 2:**
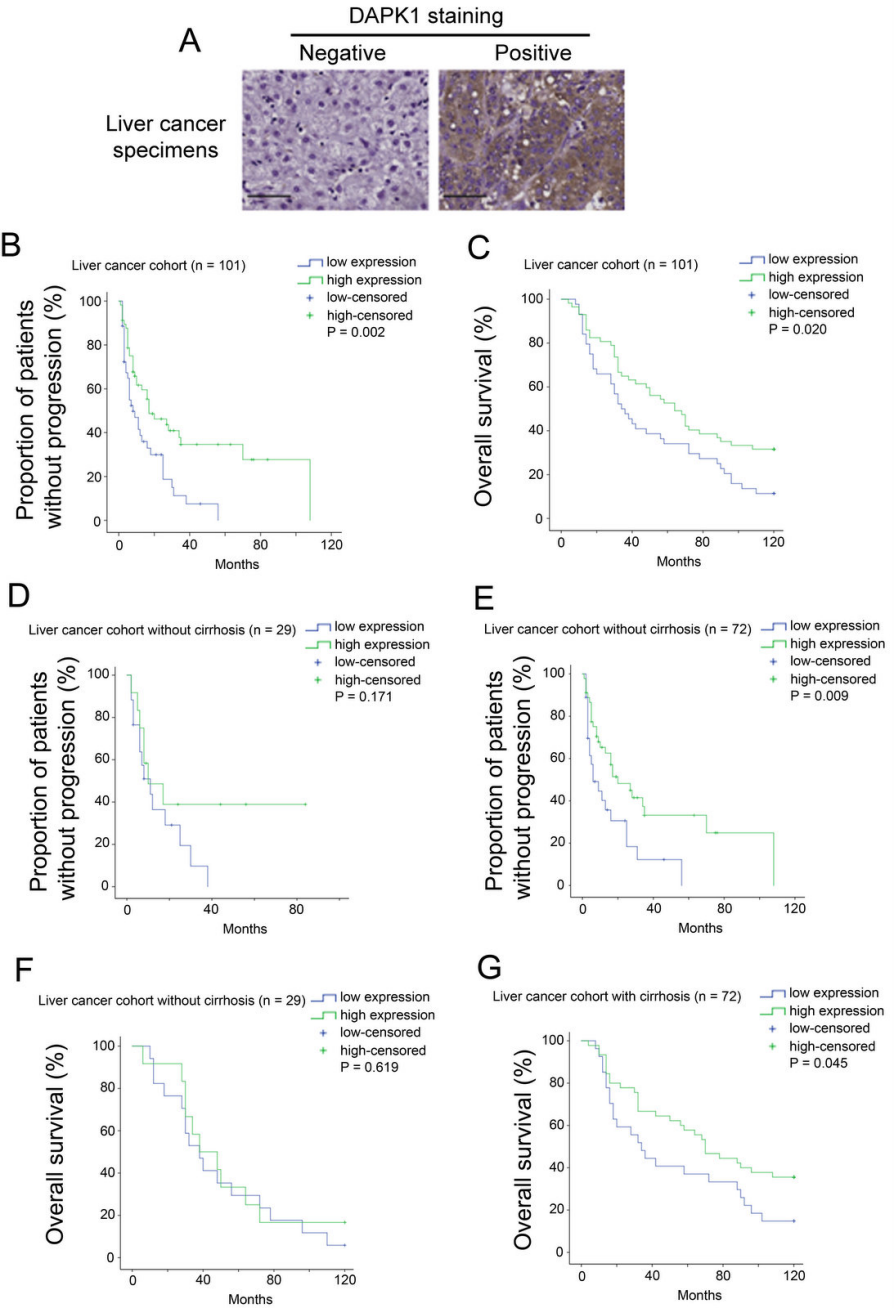
Protein expression of DAPK1 in liver cancer specimens. (A) Representative immunohistochemistry images for liver cancer specimens stained negative and positive for DAPK1. Scale bar = 100 µm. (B) Kaplan–Meier time to progression curves for patients with different types of staining for DAPK1 in the in-house liver cancer cohort. (C) Kaplan–Meier overall survival curves for patients with different types of staining for DAPK1 in the in-house liver cancer cohort. (D) Kaplan–Meier time to progression curves for patients with different types of staining for DAPK1 in patients without cirrhosis. (E) Kaplan–Meier time to progression curves for patients with different types of staining for DAPK1 in patients with cirrhosis. (F) Kaplan–Meier overall survival curves for patients with different types of staining for DAPK1 in patients without cirrhosis. (G) Kaplan–Meier overall survival curves for patients with different types of staining for DAPK1 in patients with cirrhosis.

As DAPK1 mRNA expression is a prognostic marker for liver cancer patients, it is interesting to also look at the prognostic significance of DAPK1 protein expression to confirm the results obtained for mRNA studies. In view of this, immunohistochemical staining of DAPK1 was performed in 101 liver cancer specimens from 101 individual patients with various clinico-pathological parameters available. As shown in Fig. 2A, DAPK1 staining was mainly cytoplasmic, and there were 57 out of 101 (56%) specimens stained positive for DAPK1, while the rest 44 out of 101 (44%) specimens stained negative for DAPK1.

In the 44 patients with negative DAPK1 staining, 35 patients (80%) had progression, but for those 57 patients with positive DAPK1 staining, only 34 patients (60%) had progression. The mean time to progression of patients with negative DAPK1 staining was 15.3 months (95% CI [10.2–20.4] months), which was significantly shorter than the mean time to progression of 43.1 months (95% CI [29.7–56.5] months) for those patients who stained positive for DAPK1 (log-rank test, *p* = 0.002; Fig. 2B). Patients with positive staining of DAPK1 also had a significantly longer overall survival time, with a mean survival time of 33.9 months (28.4–39.3 months) compared to 25.4 months (95% CI [19.8–31.1] months) for those patients with a negative staining of DAPK1 (log-rank test, *p* = 0.02; Fig. 2C).

Interestingly, the significant association between DAPK1 staining and time to progression was not observed in patients without cirrhosis (*p* = 0.171; Fig. 2D), but in the subgroup of patients with cirrhosis, positive staining of DAPK1 was associated with significantly longer time to progression (mean time to progression = 42.0 months, 95% CI [27.1–56.9] months) compared to those patients with negative staining of DAPK1 (mean time to progression = 15.9 months, 95% CI [8.4–23.4] months, *p* = 0.009; Fig. 2E). Similar results were obtained for overall survival. In patients without cirrhotic liver, the association between DAPK1 staining and survival was not significant (*p* = 0.619; Fig. 2F), but in patients with cirrhotic liver, positive DAPK1 staining was significantly associated with a longer survival time (mean = 35.8 months, 95% CI [27.1–37.1] months) compared to those patients who had a negative staining of DAPK1 (mean survival time = 26.1 months, 95% CI [18.4–33.8] months, *p* = 0.045; Fig. 2G). These results suggest that the cirrhosis may be a factor that could influence the prognostic significance of DAPK1 expression in liver cancer.

### DAPK1 is an independent prognostic marker in liver cancer

To investigate whether DAPK1 is an independent prognostic marker in liver cancer, univariate and multivariate cox-regression analyses were performed. As shown in Table 2A, univariate cox-regression analysis showed that Sex, Age, Tumor Size and DAPK1 Staining Status were significantly associated with time to progression and these four factors were independent prognostic predictors for time to progression in the forward stepwise multivariate cox-regression analysis. For overall survival, Age, Tumor Size, Differentiation Status and DAPK1 Staining Status were significantly associated with survival in univariate cox-regression analysis, while only Age, Tumor Size and DAPK1 Staining Status were significantly associated with overall survival in the forward stepwise multivariate cox-regression analysis (Table 2B). These results suggest that DAPK1 is a novel independent prognostic marker in liver cancer.

**Table 2:** Cox regression. (A) Cox-regression analysis of time to progression. (B) Cox-regression analysis of overall survival.

Clinicopathological variables	Univariate analysis		Multivariate analysis	
	Hazard ratio (95% CI)	*p*-value	Hazard ratio (95% CI)	*p*-value
**(A) Cox-regression analysis of time to progression**				
Sex	2.679 (1.154–6.223)	0.022	2.861 (1.210–6.762)	0.017
Age	0.964 (0.941–0.988)	0.004	0.961 (0.937–0.985)	0.002
Tumor size	1.061 (1.008–1.115)	0.022	1.063 (1.008–1.121)	0.025
Tumor number	1.088 (0.701–1.689)	0.707		
Cirrhosis status	0.909 (0.678–1.218)	0.522		
Differentiation status	0.770 (0.541–1.097)	0.770		
**DAPK1 staining status**	**0.486 (0.299 = 0.790)**	**0.004**	**0.479 (0.291–0.791)**	**0.004**
**(B) Cox-regression analysis of overall survival**				
Sex	1.400 (0.721–2.722)	0.320		
Age	0.969 (0.948–0.992)	0.007	0.976 (0.954–0.997)	0.027
Tumor size	1.077 (1.029–1.128)	0.001	1.077 (1.030–1.127)	0.001
Tumor number	1.021 (0.644–1.618)	0.931		
Cirrhosis status	0.841 (0.636–1.113)	0.227		
Differentiation status	0.675 (0.484–0.941)	0.021		
**DAPK1 staining status**	**0.597 (0.382–0.932)**	**0.023**	**0.604 (0.384–0.951)**	**0.030**

### The role of b-catenin in DAPK1 expression

**Figure 3:**
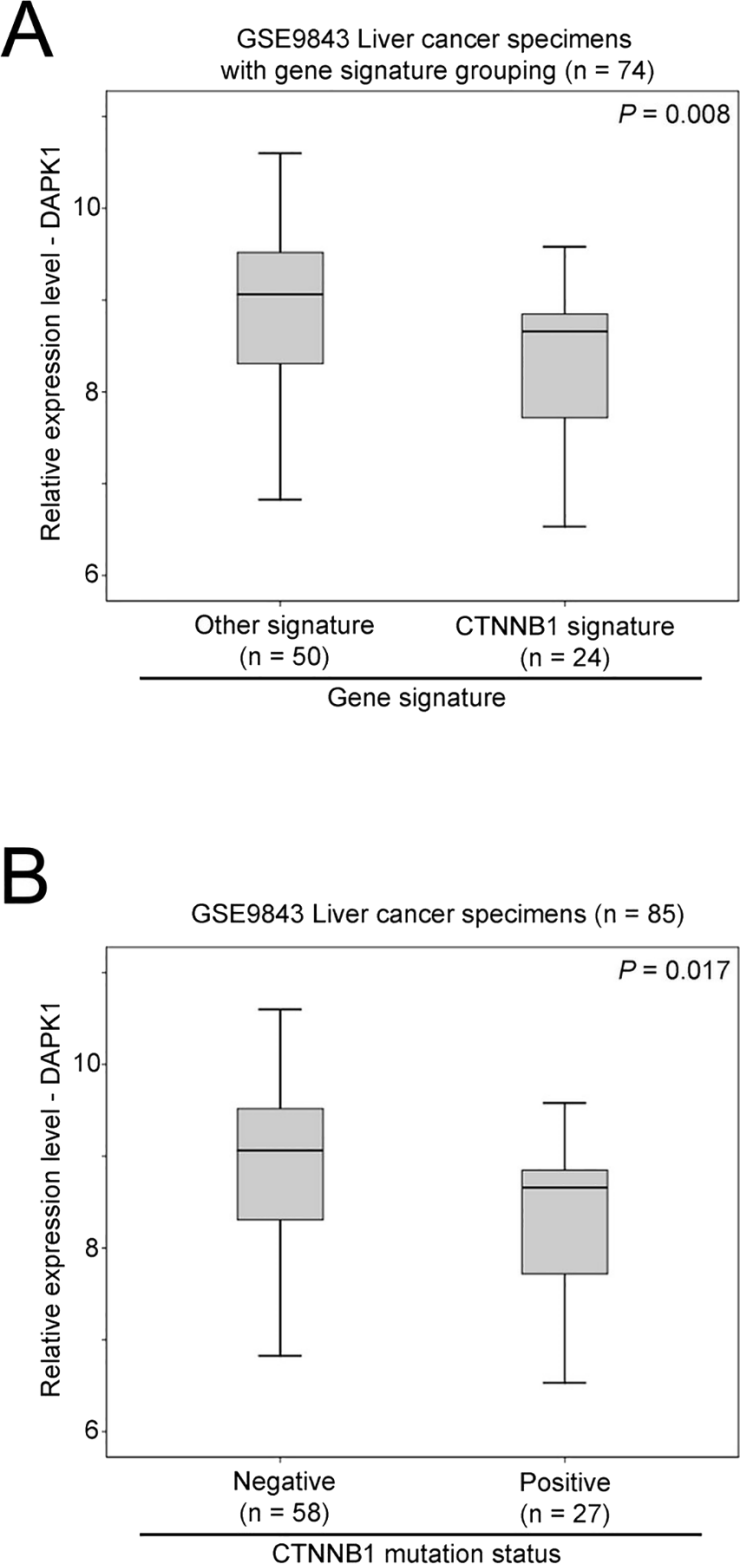
The association between beta-catenin and DAPK1 expression. (A) A box plot showing DAPK1 mRNA expression in liver cancer specimens with different types of gene signature. (B) A box plot showing DAPK1 mRNA in liver cancer specimens with different mutational status of beta-catenin.

To investigate what factor may possibly lead to dysregulation of DAPK1 in liver cancer, we identified another dataset, GSE9843, which has an annotated gene signature for 74 liver cancer specimens. From this dataset, we found that the CTNNB1 subtype of liver cancer had a significantly lower expression of DAPK1 compared to other subtypes of liver cancer (Mann–Whitney *U* test, *p* = 0.008; Fig. 3A). Interestingly, patients with mutated b-catenin status also had a significantly lower expression of DAPK1 compared to those patients who did not have mutated b-catenin (Mann–Whitney *U* test, *p* = 0.017; Fig. 3B). These results suggest that the b-catenin pathway may be an upstream regulator of DAPK1 in liver cancer progression.

### Identification of DAPK1 co-regulated genes in liver cancer

**Figure 4:**
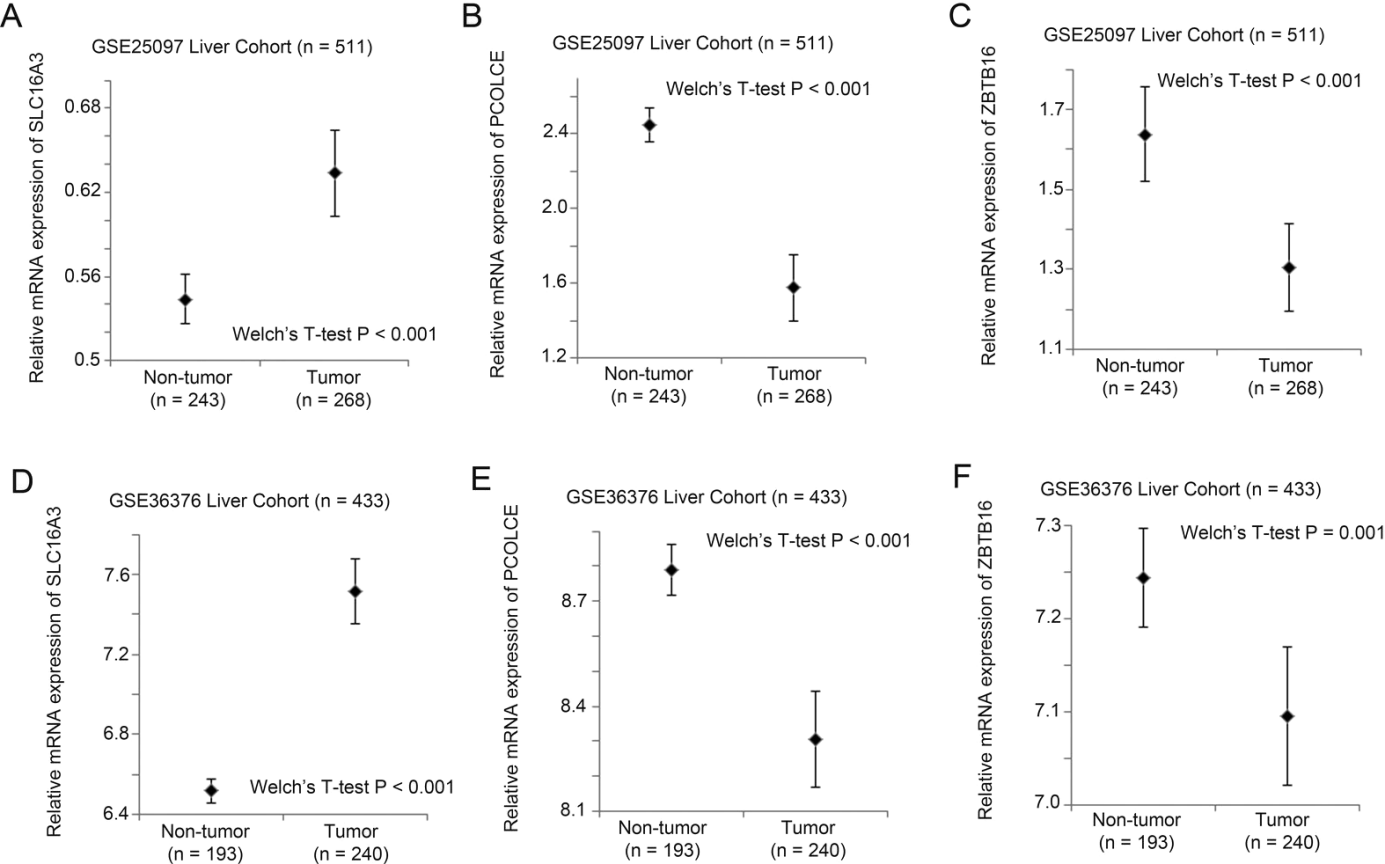
The expression levels of SLC16A3, PCOLCE and ZBTB16 in non-tumor and tumor liver specimens. Error plots for the mRNA expression of (A) SCL16A3, (B) PCOLCE and (C) ZBTB16 in GSE25097 and (D) SCL16A3, (E) PCOLCE and (F) ZBTB16 in GSE36376 liver patient cohorts.

Comparing liver specimens with differential DAPK1 expression, we have identified top five genes that are co-regulated in the two large liver patient cohorts (GSE25097 and GSE36376). DAPK1 expression level was positively and significantly correlated with the expression level of IRF2 (*r* = 0.308, *p* < 0.001), IL7R (*r* = 0.234, *p* < 0.001), PCOLCE (*r* = 0.333, *p* < 0.001) and ZBTB16 (*r* = 0.269, *p* < 0.001) in GSE25097. Similar results were obtained in GSE36376 in which DAPK1 expression level was significantly positively correlated with the expression level of IRF2 (*r* = 0.189, *p* < 0.001), IL7R (*r* = 0.219, *p* < 0.001), PCOLCE (*r* = 0.311, *p* < 0.001) and ZBTB16 (*r* = 0.190, *p* < 0.001). On the other hand, DAPK1 expression level was significantly negatively correlated with that of SLC16A3 in both GSE25097 (*r* =  − 0.301, *p* < 0.001) and GSE36376 (*r* =  − 0.295, *p* < 0.001). We further investigated how these genes may contribute to liver carcinogenesis by looking at the expression levels of these genes in non-tumor and cancerous liver specimens. As shown in Fig. 4, SLC16A3 expression was significantly upregulated in liver cancer specimens compared to non-tumor liver specimens in both GSE25097 (*p* < 0.001; Fig. 4A) and GSE36376 (*p* < 0.001; Fig. 4D), while POLOCE (GSE25097: *p* < 0.001; Fig. 4B, GSE36376: *p* < 0.001; Fig. 4E) and ZBTB16 (GSE25097: *p* < 0.001; Fig. 4C, GSE36376 *p* = 0.001; Fig. 4F) expression levels was significantly lower in liver cancer specimens compared to non-tumor specimens. These results suggest that SLC16A3 may promote while POLOCE and ZBTB16 may suppress liver carcinogenesis.

### Identification of small molecule that suppress DAPK1 and its co-regulated genes

Since DAPK1 expression was correlated with expression of SCL16A3, POLOCE and ZBTB16, while the expression levels of these genes were significantly differentially expressed in non-tumor and tumorous liver specimens. These genes were input in connectivity mapping for identification of small molecules that may reverse the expression patternscorrelated with liver carcinogenesis. In this exercise, we have identified amcinonide and sulpiride as potential small molecules that may inhibit DAPK1-mediated liver carcinogenesis.

## Discussion

In this study, we demonstrated, for the first time that, DAPK1 was down-regulated in liver cancer compared to the non-tumor liver specimens. We also showed for the first time that DAPK1 expression was a predictor of patient survival in both mRNA and protein levels. Importantly, DAPK1 expression was an independent predictor for both time to progression and overall survival in our Chinese liver cancer patient cohort, suggesting that DAPK1 plays an important in the carcinogenesis and progression of liver cancer and is a novel prognostic marker for liver cancer patients.

DAPK1 is a protein that could function either as oncogene or tumor suppressor gene in different cellular context (Steinmann et al., 2015). In the present study, we have observed in three independent microarray datasets consisting of 1,105 liver specimens, and an in-house liver cancer patient cohort, that DAPK1 may act as a tumor suppressor gene and its high level expression may be a favorable prognostic marker in liver cancer in both mRNA and protein levels. The results suggest that DAPK1 dysregulation may be an early event in liver cancer development. Indeed, majority of cirrhosis specimens also had a low level expression of DAPK1 compared to the healthy or adjacent non-tumor specimens, further supporting the hypothesis that DAPK1 down-regulation may happen early in the development of liver cancer.

Although our findings have provided evidence on the prognostic significance of DAPK1 mRNA in liver cancer, there is still little evidence showing how DAPK1 mRNA is regulated. As methylation of DAPK1 was not detected in liver cancer specimens (Yu et al., 2002), it is highly possible that the dysregulation of DAPK1 mRNA expression was at its transcriptional level. In view of this, we have further identified a published microarray dataset with annotated gene signature. We have shown that DAPK1 was significantly down-regulated in the CTNNB subtype of liver cancer, and importantly in liver cancer with a b-catenin mutation, the expression of DAPK1 mRNA was significantly lower compared to those liver cancer with wild-type b-catenin. Our results suggest that the Wnt/b-catenin pathway may play a role in DAPK1 down-regulation in liver cancer. The Wnt/b-catenin pathway, when activated, is sufficient to cause hepatocellular carcinoma and hepatoblastoma, suggesting that this pathway plays an important role in liver carcinogenesis (Mokkapati et al., 2014). In gastric cancer cells, both b-catenin and DAPK1 expression was regulated by S100P, which is an oncogenic factor in gastric cancer (Zhang et al., 2014). The mechanistic insights for how DAPK1 is regulated by b-catenin in liver cancer may result in novel therapeutic approach in treatment of liver cancer. Liver cirrhosis is commonly present in most patients with hepatocellular carcinoma with limited effective treatments, of which liver transplantation is one strategy to cure both diseases (Granito & Bolondi, 2017). As demonstrated in our China patient cohort, we found that the association between DAPK1 expression and survival was significant in liver cancer patients who had also cirrhosis implies that DAPK1 may be a potential therapeutic target for this particular group of patients.

In the current study, we have also identified few genes that are co-regulated with DAPK1, especially for SLC16A3, PCOLCE and ZBTB16, since the expression of these genes were dysregulated in liver cancer. Little is known how these genes contribute to liver carcinogenesis. SLC16A3, encoding a monocarboxylate transporter 4 protein, has been shown to be a downstream factor of TSTA3 immune response-mediated metabolism coupling cell cycle to postreplication repair in no-tumor hepatitis/cirrhotic tissues (Wang et al., 2012). PCOLCE (Procollagen C-Endopeptidase Enhancer) has been shown to be the strongest in its correlation with the liver fibrosis phenotype (Ippolito et al., 2016). ZBTB16, also named as PLZF, has previously been shown to be down-regulated in hepatocellular carcinoma, which supports our current study that ZBTB16 was down-regulated in liver cancer specimens compared to non-tumor liver specimens (Hui et al., 2015). PIN1 has been shown to contribute to hepatic carcinogenesis (Pang et al., 2006), while targeting PIN1 has been suggested to be a promising therapeutic approach for hepatocellular carcinoma . Although we have not looked into the association between DAPK1 and PIN1, in the two available liver cancer cohort identified in GEO database, the expression of DAPK1 and PIN1 was significantly inversely correlated, suggesting that DAPK1, act as a liver cancer suppressor, and PIN1, act as a liver cancer oncogenic factor, may interact with each other, though the mechanism is currently unclear, to exert their function in liver cancer.

By connectivity mapping, we have identified sulpiride as one of the potential small molecules that could reverse DAPK1 and its co-regulated genes expression pattern in liver cancer, which may have potential therapeutic value in liver cancer, especially for those with reduced DAPK1 expression. As sulpiride has been shown to inhibit gastric carcinogenesis (Tatsuta et al., 1991) as well as endometrial carcinogenesis (Taketa et al., 2016) in rats, it would be interesting to further test how the potential of sulpiride in inhibiting liver carcinogenesis and how DAPK1 may be involved in future study.

In conclusion, the present study has shown that DAPK1 donwregulation happens early in liver cancer development and confers poor prognosis in liver cancer patients. The down-regulation of DAPK1 is at its mRNA level. Further investigation is required to confirm the role of the Wnt/b-catenin pathway in DAPK1 mRNA down-regulation.

##  Supplemental Information

10.7717/peerj.3568/supp-1 Supplemental Information 1 GSE9843 dataset Raw data.

10.7717/peerj.3568/supp-2 Supplemental Information 2 GSE25097
Raw data.

10.7717/peerj.3568/supp-3 Supplemental Information 3 GSE36376
Raw data.

10.7717/peerj.3568/supp-4 Supplemental Information 4 GSE76427
Raw data.

10.7717/peerj.3568/supp-5 Supplemental Information 5 DAPK staining dataset Raw data.

## Additional Information and Declarations

### Data Availability

The following information was supplied regarding data availability: The raw data has been provided as Supplementary Files.
